# Sleep monitoring challenges in patients with neurocognitive disorders: A cross‐sectional analysis of missing data from activity trackers

**DOI:** 10.1002/hsr2.608

**Published:** 2022-04-26

**Authors:** Manan Ahuja, Shailee Siddhpuria, Christina Reppas‐Rindlisbacher, Eric Wong, Jessica Gormley, Justin Lee, Christopher Patterson

**Affiliations:** ^1^ Division of Geriatric Medicine, Department of Medicine McMaster University Hamilton Ontario Canada; ^2^ Department of Undergraduate Medicine University of British Columbia Vancouver British Columbia Canada; ^3^ Division of Geriatric Medicine, Department of Medicine University of Toronto Toronto Ontario Canada; ^4^ Division of Plastic Surgery, Department of Surgery McMaster University Hamilton Ontario Canada

**Keywords:** activity monitor, delirium, dementia, hospitalized older adults, neurocognitive disorder

## Abstract

**Background and Aims:**

Activity monitors, such as Fitbits®, are being used increasingly for research purposes and data have been validated in healthy community‐dwelling older adults. Given the lack of research in older adults with neurocognitive disorders, we investigated the consistency of sleep data recorded from a wrist‐worn activity monitor in this population.

**Methods:**

Fitbit® activity monitors were worn by hospitalized older adults as part of a parent study investigating sleep and step count in patients recovering from hip fracture surgery in a tertiary care academic hospital in Hamilton, Canada between March 2018 and June 2019. In this secondary analysis, we compared the proportion of missing sleep data between participants with and without a neurocognitive disorder and used a multivariable model to assess the association between neurocognitive disorder and missing sleep data.

**Results:**

Of 67 participants included in the analysis, 22 had a neurocognitive disorder (median age: 86.5 years). Sleep data were missing for 47% of the neurocognitive disorder group and 23% of the non‐neurocognitive disorder group. The presence of a neurocognitive disorder was associated with an increased likelihood of missing sleep data using the Fitbit® activity monitors (adjusted odds ratio: 3.41; 95% confidence interval: 1.06–11.73, *p* = 0.04).

**Conclusion:**

The inconsistent nature of sleep data tracking in hospitalized older adults with neurocognitive disorders highlights the challenges of using interventions in patient populations who are often excluded from validation studies. As opportunities expand for activity monitoring in persons with neurocognitive disorders, novel technologies not previously studied in this group should be used with caution.

## 1 INTRODUCTION

Poor sleep is common in hospitalized older adults. It is experienced by up to 47% of patients on medical units^1^ and up to 67% of patients hospitalized for psychiatric conditions.^2^ Patients admitted to general medical wards report an average of 1.5 less hours of sleep and have longer daytime naps compared to their home environments.^3^ Poor sleep is a consequence of several factors, including acute illness, uncontrolled pain, external environment stimuli, and overnight interruptions causing frequent arousals.^4^, ^5^ In the general population, poor sleep quality affects neurocognitive health, increasing the risk of dementia and anxiety.^6^, ^7^ Poor sleep experienced by hospitalized older adults recovering from surgery has been associated with delirium^8^ in the intensive care unit and other hospital settings.^9^, ^10^ Daytime fatigue from poor sleep may result in immobility during the day,^11^ impacting participation in care plans such as physiotherapy.

Polysomnography (PSG), the gold standard sleep measurement method, is not commonly used in inpatient clinical settings, due to its high‐cost and invasiveness.^12^ However, developments in wrist worn activity monitor technology have allowed for easier collection of sleep data in hospitalized patients. Activity monitors are nonintrusive and can collect data continuously over many days. Most commercial activity monitors such as Fitbits® recognize sleep onset and periods of wakefulness using an algorithm of movement detection and heart rate monitoring.^13^ Fitbit activity monitors have been compared to PSG in outpatient settings with healthy individuals and demonstrate acceptable agreement in terms of total sleep time, sleep efficiency,^14^ sensitivity (ability to detect true sleep) and accuracy (ability to detect both true sleep and true wakefulness).^15^ Although wrist activity monitors provide an alternate method to measure sleep for clinical and research and there is fair agreement in healthy populations, their ability to measure sleep in hospitalized patients with neurocognitive disorders remains untested.^16^

Our objective was to compare the consistency of activity monitors to record and measure sleep between study participants with and without neurocognitive diagnoses.

## 2 METHODS

The parent study, **R**esearching the **E**ffects of sleep on **ST**ep count d**U**ring the **P**ost‐operative period (REST‐UP), investigated the impact of sleep duration on outcomes in older adults recovering from hip fracture surgery.^8^ We included 67 patients who were 65 years of age or older and admitted with hip fracture to the orthopedic ward of a tertiary academic hospital in Hamilton, Canada. Wrist activity monitors (Fitbit® Alta 2) were applied to older adults, who provided informed consent themselves or via a substitute decision maker, after hip fracture surgery and worn for the duration of their hospital stay (or up to 14 days). We conducted an exploratory analysis of the device data from participants with neurocognitive disorders defined as: mild cognitive impairment (MCI/minor neurocognitive disorder), dementia (major neurocognitive disorder), Parkinson's disease and those who developed postoperative delirium. Preexisting diagnoses of MCI, dementia and Parkinson's disease were extracted from the chart from admission and consultation notes and postoperative delirium was extracted using the CHART‐DEL (Chart‐based Delirium Identification Instrument) method.^17^

The average proportion of sleep data that failed to be recorded from participants with these conditions was compared to data from other participants in the study. We defined failure to record sleep data as the activity monitor not tracking sleep for one or more nights. Differences in proportions were calculated using Chi‐squared test and differences in means were calculated using the *t* test. A multivariable model was created to evaluate the association between neurocognitive disorders and missing sleep data after adjusting for age, sex, and Charlson comorbidity index. All tests of significance were two‐sided and *p* values less than 0.05 were consider significant. The statistical analysis was done using R version 4.0.3. This study was approved by the Hamilton Integrated Research Ethics Board (#4300—February 2018) and was registered on clinicaltrials.gov (CT03776526). The STROBE checklist for cross‐sectional studies was reviewed to ensure items #1–22 were completed.

## 3 RESULTS

There were 22 participants (median age: 86.5 years; female: 68.2%) in the neurocognitive disorder group and 45 participants in the group without a neurocognitive diagnosis (median age: 80.0 years; female: 64.4%). The median age in the neurocognitive disorders group was higher (86.5 vs. 80.0 years, *p* = 0.03). Eighty‐two percent of participants in the neurocognitive disorder group received a diagnosis of postoperative delirium during their inpatient stay and 32% had a previous diagnosis of a neurocognitive disorder. All baseline characteristics are shown in Table 1, including postoperative mobility and living environment. The proportion of missing sleep data was higher in those with neurocognitive disorders compared to those without neurocognitive disorders (47.0% vs. 23.0%, *p* = 0.004). There was a higher mean number of missing sleep days in those with neurocognitive disorders (3.32 vs. 1.49, *p* = 0.007), though the mean number of days participants wore the Fitbit® was similar between both groups (6.86 vs. 6.89, *p* = 0.98). In the multivariable analysis (Table 2), the presence of a neurocognitive disorder was an independent predictor of missing sleep data (adjusted odds ratio [aOR]: 3.41; 95% confidence interval [Cl]: 1.06–11.73, *p* = 0.04), after adjusting for age (aOR: 1.01, 95% CI: 0.94–1.10, *p* = 0.75), female sex (aOR: 0.40, 95% CI: 0.11–1.38, *p* = 0.152), and Charlson comorbidity index (aOR: 0.88, 95% CI: 0.57–1.30, *p* = 0.57).

**Table 1:** Baseline characteristics and sleep‐related results for participants stratified by the presence of a neurocognitive disorder

Characteristics	Neurocognitive disorder (*n* = 22)	No neurocognitive disorder (*n* = 45)
Age, median (IQR)	86.5 (82.3–91.5)	80.0 (74.0–88.0)
Female, *n* (%)	16 (72.7)	29 (64.4)
Charlson comorbidity index, median (IQR)	5.5 (5.0–6.0)	5.05 (4.0–6.0)
Postoperative delirium, *n* (%)	18 (81.8)	N/A
Neurocognitive disorder (dementia), *n* (%)	7 (31.8)	N/A
Postoperative mobility, *n* (%)		
Independent	0 (0.0)	0 (0.0)
Requires assistance	20 (90.9)	43 (95.6)
Nonambulatory	2 (9.1)	1 (2.2)
Unknown	0 (0.0)	1 (2.2)
Postdischarge disposition, *n* (%)		
Community	1 (4.5)	7 (15.6)
LTC/convalescent care	7 (31.8)	2 (4.4)
Rehabilitation	11 (50)	34 (75.6)
Other	3 (13.6)	2 (4.4)
Number of days Fitbit® worn, mean (*SD*)	6.86 (3.59)	6.89 (3.28)
Mean number of missing sleep days, mean (*SD*)	3.32 (3.37)	1.49 (2.03)
Mean proportion of missing sleep days per participant, mean (*SD*)	0.47 (0.40)	0.23 (0.26)

Abbreviations: IQR, interquartile range; LTC, long‐term care; SD, standard deviation.

**Table 2:** Multivariable model of predictors of missing activity monitor sleep data

Predictor	Unadjusted OR (95% CI)	Adjusted OR (95% CI)^a^	*p* value
Neurocognitive disorder present	3.06 (1.03–9.31)	3.41 (1.06–11.73)	0.04^b^
Age	1.02 (0.96–1.09)	1.01 (0.94–1.09)	0.75
Sex (female)	0.55 (0.18–1.63)	0.39 (0.11–1.37)	0.15
Charlson comorbidity index	1.02 (0.73–1.45)	0.89 (0.57–1.30)	0.57

Abbreviations: CI, confidence interval; OR,odds ratio.

^a^ Adjusted for age, sex and Charlson comorbidity index.

^b^ Statistically significant value.

## 4 DISCUSSION

Study participants with neurocognitive disorders had more than three times the odds of having missing sleep data compared to participants without neurocognitive disorders, indicating that sleep may not be measured reliably by activity monitors in all populations. There are a number of possible explanations for this finding. First, participants with neurocognitive disorders were more likely to remove or dislodge the activity monitors at night. The removal of activity monitors by these patients has been previously reported^18^ and presents a challenge to measuring sleep in this population, however, we cannot confirm whether this occurred in our participants as they were not constantly monitored at night. Second, in patients with neurocognitive disorders, the Fitbits® may have had trouble establishing a “baseline period,” which helps calibrate the device to measure sleep accurately. A recent systematic review investigating actigraphy in patients with Alzheimer's dementia, recommended a minimum 7‐day period to establish an accurate baseline of sleep characteristics before collecting data.^19^ Given the limitations of our inpatient study, we were unable to spend several days establishing a “baseline period” for patients. As the Fitbits® were usually worn for less than 7 days due to short hospital stays, it is unlikely that baseline sleep characteristics were adequately established. Last, since wrist activity monitors track sleep based on heart rate and physical movement,^13^ any increased movement during sleep could be associated with inaccurate recording of sleep. Abnormal movement during sleep is especially common in patients with neurocognitive disorders such as Parkinson's disease,^20^ Lewy body dementia,^21^ and hyperactive and mixed delirium.^22^ As a result, increased movements at night in the neurocognitive disorder group could have contributed to the disproportionate missing sleep data between the two groups.

Older adults, especially those with multimorbidity, including neurocognitive impairment, are often excluded from validation or efficacy trials.^16^, ^23^ While it is important to ensure the safety of patients in clinical trials, it is sometimes difficult to assess the generalizability of new interventions, including activity monitors such as Fitbits®, in this population. While this study demonstrated data collection inaccuracies in this population, it is important to continue evaluating the use of activity monitors to measure sleep in patients with neurocognitive disorders. Expanding our ability to measure sleep quality and duration in this patient population is an important area of future research in settings of care such as hospitals, where sleep is known to be insufficient. Future investigation should compare the accuracy of wearable sleep technology with the gold‐standard method of PSG in this population specifically. This will help address the questions raised from our study, which include whether poor sleep collection from wearable wrist activity monitors is due to abnormal sleep rhythms or due to physical restlessness among patients with neurocognitive disorders. Finally, alternate methods of monitoring sleep in the inpatient setting could be further investigated with bed sensor technology, which measures sleep through ballistocardiography. This technology could be promising method of measuring sleep for patients with neurocognitive disorders as it may better capture restlessness and awake movements in bed and are is less prone to missing data.^24^

There are study limitations worth noting. First, we were unable to compare the data collected from the Fitbit® Alta 2 with the gold‐standard method of PSG. As a result, the reason for the disproportionate missing sleep data cannot be fully elucidated. Second, the sample size of this analysis is small (*n* = 67), which is further divided between two groups, so our sample is unlikely to be representative of older adults hospitalized with neurocognitive disorders. The small sample size also precludes subgroup analysis of different neurocognitive disorders (i.e., dementia, delirium, etc.), which may have different patterns of missing sleep. Finally, while we presume that patients with delirium may have removed their activity monitors at night, however this could not be verified.

In conclusion, in this study of hospitalized older adults recovering from hip fracture surgery, sleep data measurement using wrist activity monitors were inconsistent in participants with neurocognitive disorders. This highlights the challenges of using devices that have been validated for use in healthy adults and applying them to older adults with multimorbidities. Alternate methods may be required to reliably and accurately evaluate sleep in future research investigations involving this patient population.

## AUTHOR CONTRIBUTIONS

*Conceptualization*: Manan Ahuja, Shailee Siddhpuria, Christina Reppas‐Rindlisbacher, Eric Wong, Jessica Gormley, Justin Lee, Christopher Patterson. *Data curation*: Manan Ahuja. *Formal analysis*: Eric Wong, Manan Ahuja. *Funding acquisition*: Christina Reppas‐Rindlisbacher. *Investigation*: Manan Ahuja, Shailee Siddhpuria, Christina Reppas‐Rindlisbacher, Eric Wong, Jessica Gormley, Justin Lee, Christopher Patterson. *Methodology*: Manan Ahuja, Shailee Siddhpuria, Christina Reppas‐Rindlisbacher, Eric Wong, Jessica Gormley, Justin Lee, Christopher Patterson. *Resources*: Manan Ahuja, Shailee Siddhpuria, Christina Reppas‐Rindlisbacher, Eric Wong, Jessica Gormley, Justin Lee, Christopher Patterson. *Supervision*: Christina Reppas‐Rindlisbacher, Eric Wong, Justin Lee, Christopher Patterson. *Visualization and Writing—original Draft Preparation*: Manan Ahuja. *Writing—review and editing*: Shailee Siddhpuria, Christina Reppas‐Rindlisbacher, Eric Wong, Jessica Gormley, Justin Lee. *Access to all of the data*: Christopher Patterson. *Manuscript's preparation, revision, and final approval of version to be published*: All authors.

## CONFLICTS OF INTEREST

The authors declare no conflicts of interest.

## TRANSPARENCY STATEMENT

The lead author, Dr. Manan Ahuja affirms that this manuscript is an honest, accurate, and transparent account of the study being reported; that no important aspects of the study have been omitted; and that any discrepancies from the study as planned (and, if relevant, registered) have been explained. Dr. Ahuja had full access to all of the data in this study and takes complete responsibility for the integrity of the data and the accuracy of the data analysis.

## Data Availability

The data that support the findings of this study are available on request from the corresponding author. The data are not publicly available due to privacy or ethical restrictions.
